# The Effect of Exposure on the Autogenous Self-Healing of Ordinary Portland Cement Mortars

**DOI:** 10.3390/ma12233926

**Published:** 2019-11-27

**Authors:** Magdalena Rajczakowska, Karin Habermehl-Cwirzen, Hans Hedlund, Andrzej Cwirzen

**Affiliations:** 1 Building Materials, Department of Civil, Environmental and Natural Resources Engineering, Luleå University of Technology, 97187 Luleå, Sweden; karin.habermehl-cwirzen@ltu.se (K.H.-C.); hans.hedlund@ltu.se (H.H.); andrzej.cwirzen@ltu.se (A.C.); 2 Skanska, Warfvinges väg 25, 112 74 Stockholm, Sweden

**Keywords:** autogenous self-healing, cementitious materials, cracking, exposure, microstructure, calcium phosphate

## Abstract

Exposure conditions are critical for the autogenous self-healing process of Portland cement based binder matrixes. However, there is still a significant lack of fundamental knowledge related to this factor. The aim of this paper was to investigate and understand the effects of various potentially applicable curing solutions on the efficiency of the crack closure occurring both superficially and internally. Four groups of exposures were tested, including exposure with different water immersion regimes, variable temperatures, application of chemical admixtures, and use of solutions containing micro particles. The self-healing process was evaluated externally, at the surface of the crack, and internally, at different crack depths with the use of optical and scanning electron microscopes (SEM). The phase identification was done with an energy dispersive spectrometer combined with the SEM. The results showed very limited self-healing in all pure water-based exposures, despite the application of different cycles, temperatures, and water volumes. The addition of a phosphate-based retarding admixture demonstrated the highest crack closure, both internally and externally. The highest strength recovery and a very good crack closure ratio was achieved in water exposure containing micro silica particles. The main phase observed on the surface was calcium carbonate, and internally, calcium silicate hydrate, calcium carbonate, and calcium phosphate compounds. Phosphate ions were found to contribute to the filling of the crack, most likely by preventing the formation of a dense shell composed of hydration phases on the exposed areas by crack unhydrated cement grains as well as by the additional precipitation of calcium and phosphate-based compounds. The micro sized silica particles presumably served as nucleation sites for the self-healing products growth. Changes in the chemical composition of the self-healing material were observed with a distance from the surface of the specimen.

## 1. Introduction

Exposure conditions are one of the crucial factors affecting the efficiency of the self-healing processes in cementitious materials. The autonomous systems are to some extent dependent on exposure. Full time [1,2] or at least part time water immersion [3] is a mandatory condition for the bacteria-based self-healing systems. As the presence of water might be problematic in real-life applications, the portable water-reservoirs in the form of hydrogels are used [4,5,6]. Crack widths of up to 0.5 mm are healed due to bacterial CaCO_3_ precipitation inside and on the surface of the hydrogel [5]. The crack closure efficiency increases by 50% with the use of hydrogel encapsulated bacterial spores [5]. In addition to providing water for the chemical reactions, the hydrogels serve as protection for spores against the harsh concrete environment [6]. Fresh cracks immersed for one day in calcium lactate or gluconate lead to faster healing of the bacteria based systems due to a potentially higher availability of calcium and carbonate ions [7].

While crack closure efficiency of the autogenous self-healing process in concrete depends, to a considerable extent, on the exposure conditions, there is still a very limited amount of data available on this topic. Only few studies proposed possible mechanisms to control that phenomenon (e.g., References [8,9,10]).

Constant water immersion results in effective self-healing, however, only for cracks with very limited widths [11,12,13,14,15,16]. Nevertheless, still water is more efficient than the flowing one [15]. Water promotes the formation of the healing products inside the cracks of concrete mixes with a carboxylic acid waterproofing admixture [17]. However, recently published results obtained using the X-ray microtomography suggested that mortar specimens, submerged in water, only demonstrated self-healing on the surface [18]. There is no noticeable crack closure while applying variable temperature or relative humidity [18]. Faster self-healing at higher temperatures (50 °C and 80 °C) is observed, based on the water permeability tests [19]. Crack widths below 50 μm are healable in the case of Engineered Cementitious Composites (ECC) subjected to wetting/drying cycles [20]. The self-healing process occurs fast in the beginning, but slows down after around five curing cycles [9,12,13,20,21]. Air curing [11,12] and freeze/thaw cycles [14] do not give satisfying results. Freeze/thaw cycles with deicing salt are found to be even less efficient than the water freeze/thaw cycles for ECC [22]. Seawater facilitates the self-healing of an ordinary Portland cement concrete, and promotes the closure of crack widths of up to 0.5 mm, however, the compressive strength is lowered [23].

Independently of the applied exposure, the self-healing products mostly include CaCO_3_ [11,14,15,21,22], calcium silicate hydrate (CSH) [14,21], and calcium hydroxide [22], and depend strongly on the concrete mix composition. In the case of mixes containing slag, the water-based exposure triggers precipitation of CaCO_3_ through carbonation reactions. The NaOH-based exposure promotes the formation of calcium carbonate and CSH by alkali-activation of slag hydration [8]. Exposure to Ca(OH)_2_ solution leads to a growth of CSH, ettringite, hydrogarnet, and OH-hydrotalcite [9,24]. Recently, changes in the mineralogical composition of the self-healing products with an increasing distance from the specimen’s surface are observed in the concrete exposed to seawater. Calcite tends to form close to the surface, while CSH and ettringite appear deeper inside the cracks [10,25].

To summarize, there is still a significant lack of knowledge that prevents a full understanding of the autogenous self-healing mechanism in various environmental conditions, which is crucial, especially for real-life applications. Consequently, the aim of this study is to investigate the effects of exposure to various solutions and conditions on the efficiency of the self-healing process as well as to verify the composition of the healing products forming not only on the surface, but also at different depths inside the crack.

## 2. Materials and Methods

**Figure 1:**
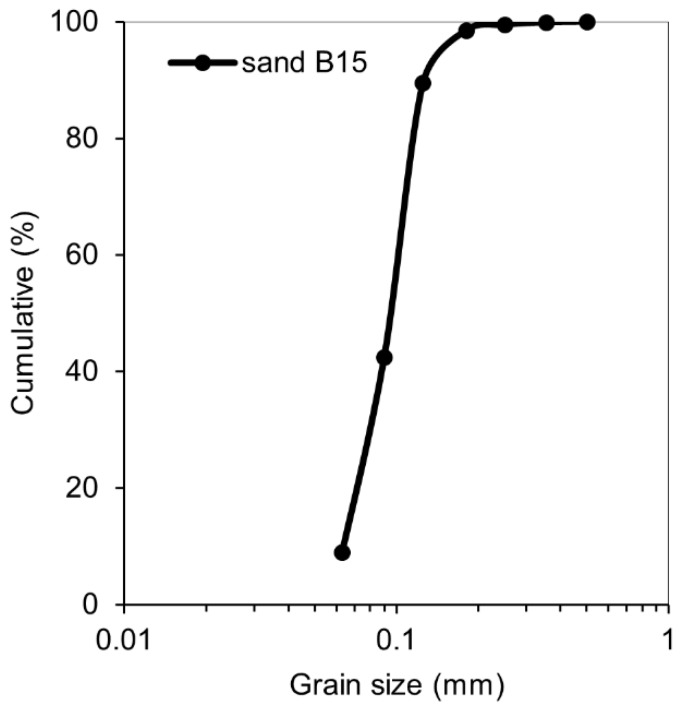
Grading curve of the sand B15 used.

Test mortars were made of Portland cement CEM I 42.5 N and had the water-to cement ratio of 0.35 and the cement to sand ratio of 1. The chemical composition and physical properties of the cement used are listed in Table 1. Sand B15 has the average particle size of 150 µm and was provided by Baskarpsand AB (Habo, Sweden) (Figure 1). The required fresh mix workability was obtained by adding 0.8 wt% (by binder) of the Sika ACE30 (Sika Sverige AB, Spånga, Sweden) superplasticizer. In order to control the crack width, 1 wt% (by binder) of Polyvinyl-alcohol (PVA) fiber was added to all the studied mixes.

Mortar beams that had dimensions of 1.2 × 1.2 × 6 cm^3^ were cast in Teflon molds without the use of demolding oil. Small vacuum mixer type Bredent was used. The beams were cracked with the use of a standard three-point bending test 7 days after casting. The test was stopped when a crack opening of around 200 µm was reached. The crack opening was assessed visually during bending, and afterwards, with an optical digital microscope. After cracking, the samples were stored in different exposures for 4 weeks. Twelve different self-healing exposure conditions were used (Table 2).

The Sika accelerator used contained 40%–60% nitrate salts and 40%–60% water. The producer described the SIKA Retarder as modified phosphates containing sodium metaphosphate (20%–30%), sodium gluconate C_6_H_11_NaO_7_ (2%–5%), and water (70%–80%). The micro silica used was Microsilica Grade 920D produced by Elkem (Oslo, Norway). The pH value of each exposure used was monitored using pH-indicator strips 30 min after the start of the experiment, and 15 h, 24 h, and every 7 days after that.

**Figure 2:**
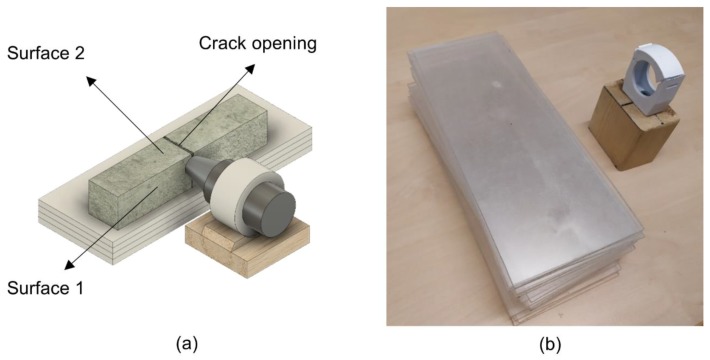
(**a**) Scheme of a designed stand; (**b**) optical microscope setup.

The self-healing efficiency was evaluated with the use of a digital optical microscope—Dino-Lite Pro AM-413T (Dino-Lite Europe, Naarden, The Netherlands). The crack opening and one of the side surfaces of the crack (Surface 1 and Surface 2) were photographed after cracking, at the end of the experiment, and after 4 weeks of exposure. Images, taken before and after healing, were studied with the use of an image processing technique in order to perform a quantitative analysis of the healing efficiency. The same crack area before and after healing was monitored with a special microscope stand (Figure 2).

**Figure 3:**
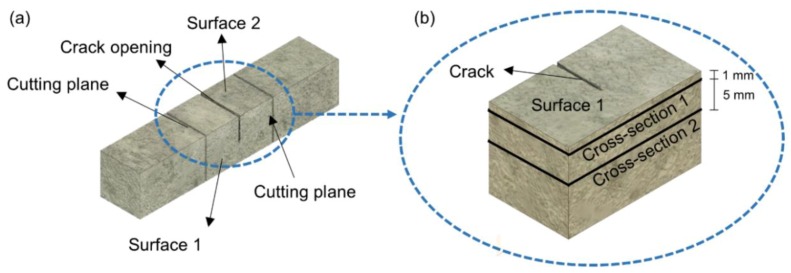
Scheme of the specimen preparation for scanning electron microscope (SEM) evaluation: (**a**) Cracked specimen with marked Surface 1 and 2; (**b**) cut fragment of the specimen to be analyzed with SEM with marked Surface 1, Cross-section 1, and Cross-section 2.

The obtained images were converted into 8-bit grayscale and were filtered with the use of a median filter with a 2-pixel kernel. Cracks were segmented from the image by applying the histogram thresholding method. ImageJ software (Version 1.51) was used for the image processing [32]. The computation of the recovery parameters was performed on binarized images of cracks, i.e., where white pixels (of value 1) depicted the crack area and black pixels (value 0) and the rest of the sample. The global crack closure ratio *C* was defined as:

(1) $$C=\frac{A_{b}-A_{h}}{A_{b}}$$

where *A_b_* and *A_h_* are the areas (sum of white pixels) of the crack before and after healing, respectively. Jeol JSM-IT100 scanning electron microscope (SEM) (JEOL Ltd., Tokyo, Japan) with Bruker energy-dispersive X-ray spectroscope (EDS) (Bruker Corporation, Billerica, MA, USA) was used to analyze the microstructure and healing products. Both secondary electron (SE) and backscatter electron (BSE) imaging modes were used. In the first step after 28 days of healing, the crack surface was evaluated to assess the presence of healing products (Surface 1). Afterwards, specimens were cut and impregnated with a low viscosity epoxy resin under a vacuum, grinded, and polished using Struers CitoVac and Lab system (Struers, Ballerup, Denmark). A set of grinding plates sprayed with diamond particles having sizes of 9, 3, and 1 µm were used as the grinding media. Two cross-sections were prepared. One was located around 1 mm below Surface 1 (Cross-section 1) and the second one was approximately 5 mm below Surface 1 in the middle of the specimen (Cross-section 2). The scheme of the specimen preparation for the SEM-EDS evaluation is shown in Figure 3.

The main aim of the SEM/EDS analysis was to determine the extent of the internal healing and to identify formed phases. The analysis included the observation of the morphology and determination of Si/Ca and Ca/P atomic ratios. In the first step, cracks were localized and healing products were photographed using various magnifications. Phases forming on Surface 1 were identified, in the preliminary stage, based on their morphology and were supported by the EDS analysis. At least three points were analyzed for each product. In the case of Cross-section 1 and 2 (Figure 3), firstly areas having similar grayscale levels, and thus, similar compositions, were selected at different crack locations. Afterwards, EDS point analysis was performed to determine the amount of the following oxides; CaO, SiO_2_, Al_2_O_3_, MgO, Na_2_O, K_2_O, SO_3_, and P_2_O_5_. Atomic ratios Si/Ca and Ca/P were calculated for each point and the mean values and standard deviations of at least three measurements were determined. The identification of Ettringite was based mainly on the observed morphology [33]. EDS point elemental analysis was not performed due to an insufficiently low resolution achievable without charging and image distortion. Instead, approximate area scanning was done, which gave an average value of the elements. Detection of sulfur was assumed to be a confirmation of the presence of this phase.

Finally, for the selected mixes, which showed the most extensive self-healing, the recovery of the flexural strength was assessed, based on testing 1.2 × 1.2 × 6 cm^3^ mortar beams. Five samples per set were investigated and average values were calculated. The mechanical strength recovery index *S* was defined as:

(2) $$S=\frac{S_{h,i}-S_{0}}{S_{0}}$$

where *S*_0_ is the flexural strength of the sample before healing. *S_h,i_* depicts the strength of the specimens healed in specific exposure *i* for 28 days. A positive sign of the strength recovery index *S* indicates an increase in strength, whereas the negative sign may suggest a lack of any effect of the healing process on the mechanical parameters.

## 3. Results

### 3.1. Crack Closure Ratio, pH Changes and Self-Healing Products

#### 3.1.1. Water Immersion Regime

**Figure 4:**
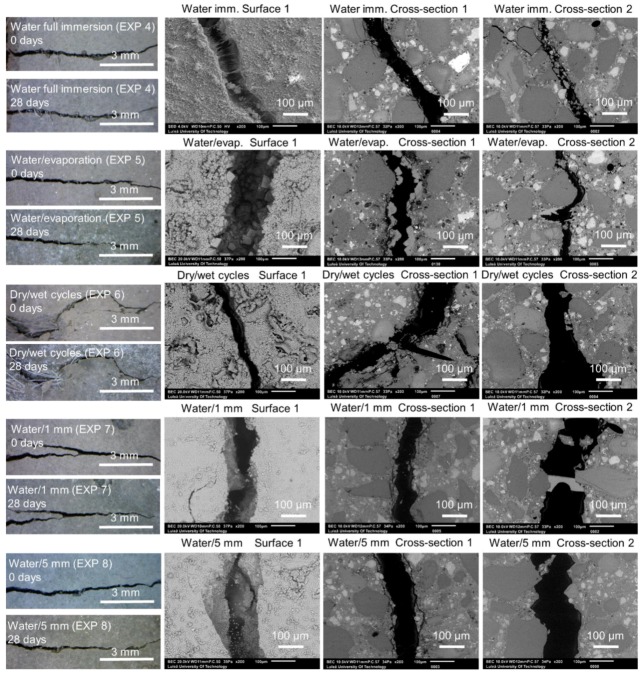
Representative images of Surface 1 from the optical microscope before (0 days) and after healing (28 days) and the SEM (BSE, 200×), as well as Cross-sections 1 and 2 (SEM BSE, 200×), of the specimens healed for 28 days in Exposures 4–8 (water immersion, water evaporation, dry/wet cycles, water/1 mm, and water/5 mm).

In general, the application of water curing using different regimes did not result in an extensive self-healing. Representative images taken with the optical microscope and with SEM are shown in Figure 4. The optical microscope images of Surface 1 were taken at the time of specimen breaking and after 28 days of healing. The SEM images were taken from Surface 1 and Cross-section 1 and 2 after 28 days of healing. The following exposures are shown: 4, 5, 6, 7, and 8 (water immersion, water evaporation, dry/wet cycles, water/1 mm, and water/5 mm). All cracks were empty after 28 days of healing.

The measured pH values of solutions in which the specimens were immersed showed a fast increase of up to 13 during the first 12 h, followed by their gradual drop for Exposures 5 (water/evaporation) and 6 (dry/wet cycles) down to pH values of 9–10 after 28 days. Exposures 4 (water immersion), 7 (water/1 mm), and 8 (water/5 mm) maintained a very high pH value throughout the entire time.

**Figure 5:**
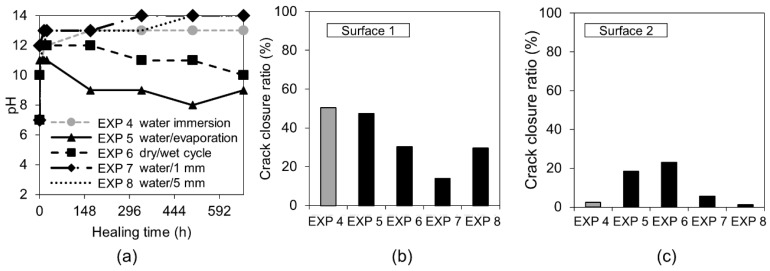
(**a**) pH changes vs. time for Exposures 4–8; crack closure ratio for Exposures 4–8; (**b**) Surface 1; and (**c**) Surface 2.

**Figure 6:**
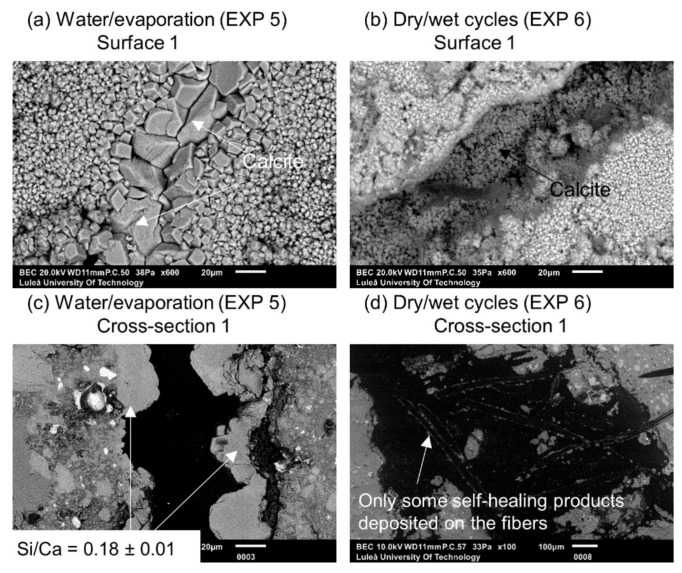
(**a**) Surface 1 Exposure 5 (SEM BSE 600×); (**b**) Surface 1 Exposure 6 (SEM BSE 600×); (**c**) Cross-section 1 Exposure 5 (SEM BSE 600×); and (**d**) Cross-section 1 Exposure 6 (SEM BSE 100×).

Exposure 4 (water immersion) and 5 (water evaporation) resulted in the highest and comparable crack closure ratio on both Surface 1 and Surface 2 (Figure 5b,c). On the contrary, in Cross-section 1, all the specimens showed hardly any healing, except for Exposure 5 (water/evaporation) where a thick layer of calcite-like product was visible with an Si/Ca ratio equal to 0.18 (Figure 6c). In the case of Exposure 5 (water/evaporation), the external crack was closed by calcite crystals when the crack opening was below 50 µm (Figure 6a). A limited healing with calcite was only observed on the surface, but not inside the crack (Figure 6b,d) in the case of the sample exposed to dry/wet cycles (EXP 7).

**Figure 7:**
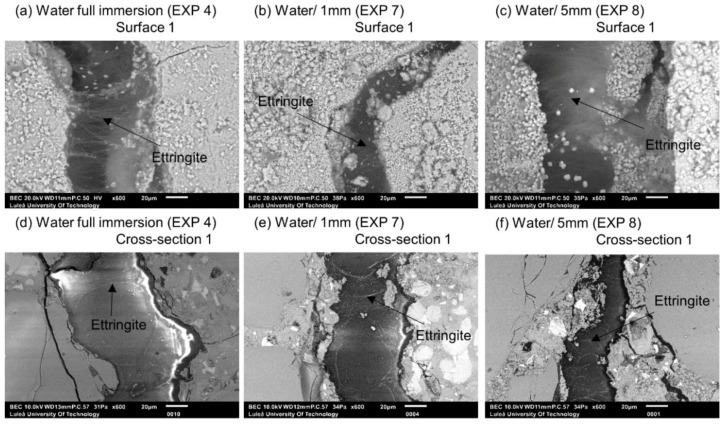
(**a**) Surface 1 Exposure 4 (SEM BE 600×), (**b**) Surface 1 Exposure 7 (SEM BSE 600×), (**c**) Surface 1 Exposure 8 (SEM BSE 600×), (**d**) Cross-section 1 Exposure 4 (SEM BSE 600×), (**e**) Cross-section 1 Exposure 7 (SEM BSE 600×), and (**f**) Cross-section 1 Exposure 8 (SEM BSE 600×).

In the case of Exposures 4 (water immersion), 7 (water/1 mm), and 8 (water/5 mm), the main phase forming on Surface 1 of the cracks was Ettringite (Figure 7). The continuous water exposure, combined with the presence of microcracks and sulfates released from unhydrated cement grains led to an internal sulfate attack with the subsequent formation of Ettringite [34]. The storing samples in water contributed to the Ettringite formation through a decrease in the pH of the pore solution caused by leaching. This led to a loss of sulfates from the CSH and to a subsequent replacement of mono-sulfate by Ettringite [33,35]. The sulfates adsorbed on the CSH surface could also contribute to the process [36].

#### 3.1.2. Temperature

**Figure 8:**
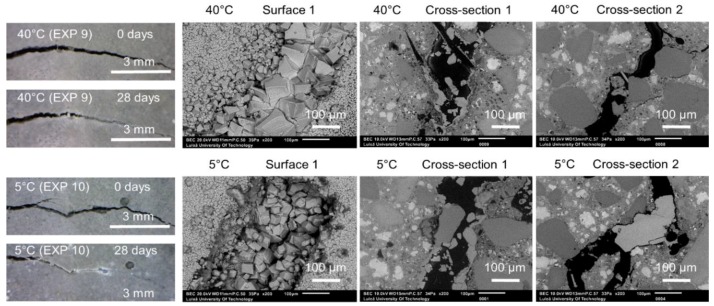
Representative images of surfaces and cross-sections of specimens healed at various temperature variation cycles (Exposure 9 and 10).

The effect of variable temperature on the healing process was verified by the application of two different temperature cycles to specimens immersed in water. The first type included 24 h of exposure to 20 °C, followed by 24 h at 40 °C (Exposure 9). The second type consisted of a storage at 20 °C for 24 h, followed by a storage at 5 °C (Exposure 10). The reference sample was stored at a constant room temperature of approximately 20 °C (Exposure 4). Figure 8 shows representative images obtained by an optical microscope from Surface 1 before and after healing. Furthermore, SEM pictures of Surface 1, Cross-section 1, and Cross-section 2 after 28 days of healing are shown for Exposures 9 and 10.

**Figure 9:**
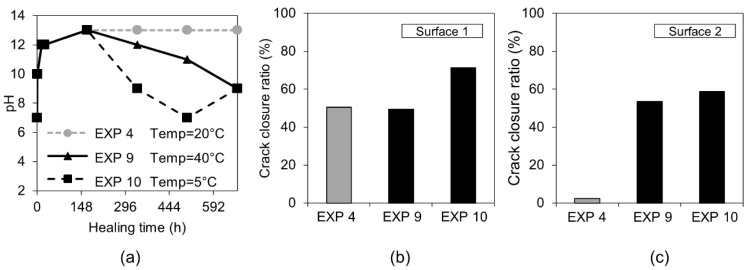
(**a**) pH changes in time for Exposures 4, 9, and 10; crack closure ratio for different temperature exposures (**b**) on Surface 1 and (**c**) on Surface 2.

The optical microscope analysis of Surface 1 did not reveal any significant differences in the observed extent of the external crack closure between different applied temperature cycles (Figure 9b). In the case of Surface 2, both higher and lower applied temperatures resulted in similar crack closures, which was, in general, slightly higher than the reference sample (Exposure 4, 20 °C) (Figure 9c). The pH values of the healing solution decreased with time for both Exposure 9 (40 °C) and Exposure 10 (5 °C), however the trend was faster at 5 °C (Figure 9a).

**Figure 10:**
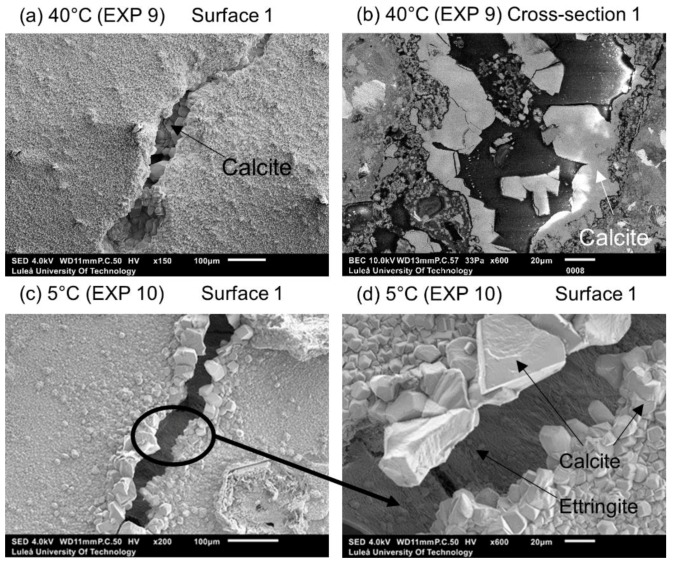
(**a**) Calcite on the surface of the crack healed in 40 °C (EXP 9, SEM SE image 150×), (**b**) calcite inside the crack (Cross-section 1) healed in 40 °C (EXP 9, SEM BSE image 800×), (**c**) Ettringite on the surface of the crack healed in 5 °C (EXP 10, SEM SE image 200×), and (**d**) Ettringite on the surface of the crack healed in 5 °C (EXP 10, SEM SE image 200×).

Calcite formed on Surface 1 in Exposure 9 (40 °C) and a mixture of calcite and Ettringite in Exposure 4 (20 °C) and Exposure 10 (5 °C) (Figure 10a,b). The Ettringite formation was evident, particularly in the low temperature exposure (Figure 10c,d), also observed by others [30,31]). None of those three exposures supported internal self-healing, with only few calcite crystals being present in Cross-sections 1 and 2 for both Exposure 9 (40 °C) and 10 (5 °C). No internal healing was observed in Exposure 4 (reference, 20 °C).

#### 3.1.3. Accelerating and Retarding Admixtures

**Figure 11:**
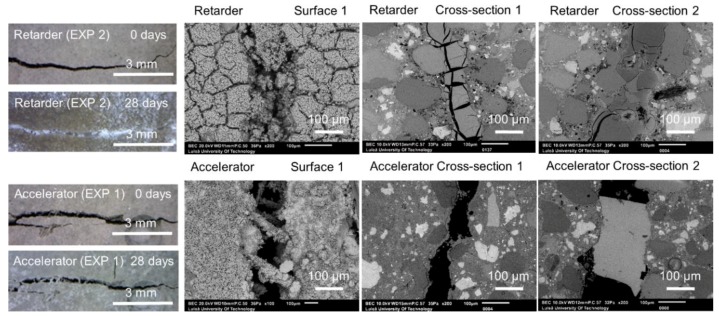
Representative images of the surface and cross-sections of the specimens healed in Exposures 2 (retarder) and 1 (accelerator).

Figure 11 shows representative optical microscope images of Surface 1, before and after healing, as well as SEM pictures of Surface 1, Cross-section 1, and Cross-section 2 obtained after 28 days of healing. Two exposures were studied in this part of the research—Exposure 1 with a retarder and Exposure 2 with an accelerator.

**Figure 12:**
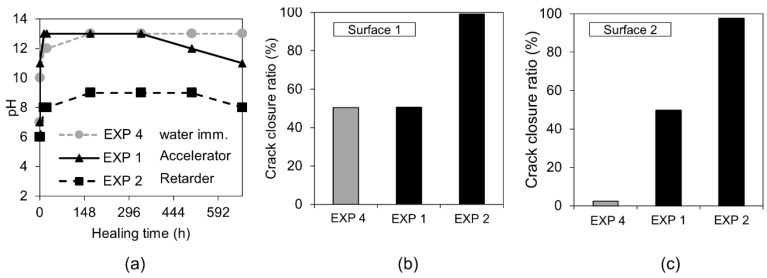
(**a**) pH changes in time for Exposures 4, 1, and 2; crack closure ratio for Exposures 5, 1, and 2 on (**b**) Surface 1 and (**c**) Surface 2.

The first visual examination done by the “naked eye” revealed that the healing products fully filled the formed crack when the retarding admixture was present in the water used in Exposure 2. Optical microscopy confirmed the presence of dense healing products in this exposure (Figure 12). Additionally, the SEM-BSE investigation showed extensive internal healing in Cross-section 1 and Cross-section 2 in Exposure 2 (retarder).

On the contrary, the accelerator used in Exposure 1 did not increase the efficiency of the healing process in comparison to the reference Exposure 4 on Surface 1. A slight crack closure was observed in Cross-Section 1 and a very extensive one in a few parts of the crack at Cross-section 2. Both cross-sections of the specimen were subjected to Exposure 1 (accelerator) and were healed better than in the case of the reference Exposure 4 (water immersion), but to a significantly lower extent in comparison with Exposure 2, which used the retarding admixture. The pH value of the solution present in the crack changed during the healing time. Interestingly, the pH of the solution containing the retarder, in the specimen that also showed the most extensive healing, was rather low, reaching only a value of nine (Figure 12a). The solution with the accelerating admixture, similar to the reference Exposure 4, achieved a pH of approximately 13 after 12 h of healing (Figure 12a). The calculated closure ratio reached 100% or nearly 100% for samples exposed to the solution with the retarder (Exposure 2) when calculated for Surface 1 and Surface 2. In the presence of the accelerator, Exposure 1 resulted in around a 50% crack closure ratio, while the reference in Exposure 4 reached a 50% crack closure on Surface 1 and 2% on Surface 2 (Figure 2).

**Figure 13:**
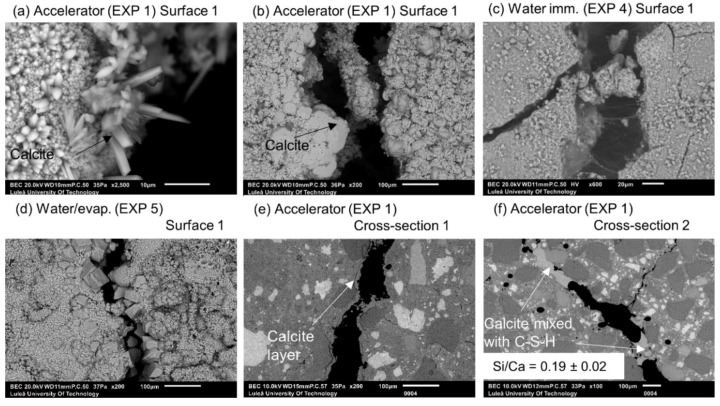
Different forms of calcite on Surface 1 of EXP 1 specimen: (**a**) SEM BE 2500×, (**b**) SEM BE 200×, and water exposures: (**c**) EXP 4 (SEM BE 600×), (**d**) EXP 5 (SEM BE 200×); (**e**) EXP 1 Cross-section 1 (visible calcite layer (SEM BE 200×)); and (**f**) EXP 1 Cross-section 2 healing products (SEM BE 100×).

The morphology of the calcium carbonate crystals found on the surface of the specimen exposed to the water solution containing the accelerator (Exposure 1) was visibly different in comparison to Exposures 4 and 5 that contained water only (Figure 13a–d). Calcite crystals are known to develop different shapes depending on the pH, i.e., elongated when there is an excess of Ca^2+^ ions and tabular in the presence of higher amounts of CO_3_^2−^ [37]. The pH effect on the calcite morphology was also observed in Reference [38]. The method used in the present research did not enable the detection of possible local variations of the pH value within the crack, which could be a direct reason for the observed different morphologies of the formed calcite. The specimen exposed to the accelerator (Exposure 1) did not demonstrate significant internal self-healing. Only few micron thick calcite layers were visible in Cross-section 1 (Figure 13c). Small deposits of calcite, possibly mixed with CSH, were detected in a few parts of the crack at Cross-section 2 (Figure 13d).

**Figure 14:**
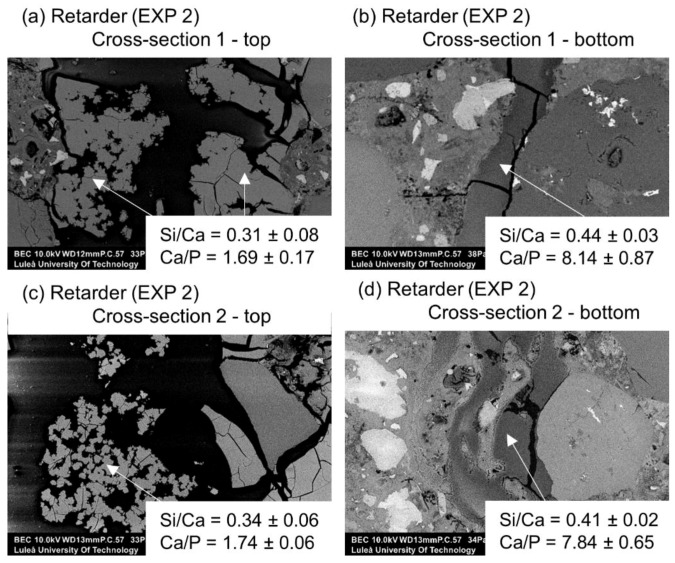
(**a**) Self-healing products at Cross-section 1 at the top of the crack—close to the crack opening (EXP 2, SEM BSE image 600×), (**b**) self-healing products at Cross-section 1 at the bottom of the crack (EXP 2, SEM BSE image 100×), (**c**) self-healing products at Cross-section 2 at the top of the crack (EXP 2, SEM BSE image 600×), and (**d**) self-healing products at Cross-section 2 at the bottom of the crack (EXP 2, SEM BSE image 600×).

**Figure 15:**
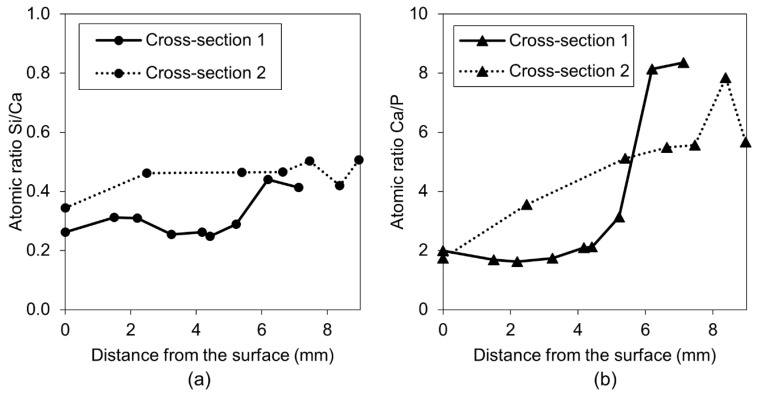
(**a**) Self-healing products Si/Ca atomic ratio vs. crack length at Cross-section 1 and 2 for Exposure 2 (retarder), and (**b**) self-healing products Ca/P atomic ratio vs. crack length at Cross-section 1 and 2 for Exposure 2 (retarder).

A more detailed SEM-EDX analysis of cracks filled with healing products in Exposure 2, which contained water with the retarder, indicated the presence of a high amount of calcium and phosphorus with traces of silicon and aluminum. The healing deposits located closer to the crack opening had lower Si/Ca and Ca/P ratios in both studied Cross-sections (Figure 14a,c). Both ratios tended to increase at higher depths, thus indicating a higher concentration of ions transported from the unhydrated cement particles. Further investigation confirmed the observed trend for both ratios for the samples in Exposure 2 (retarder) (Figure 15a,b). All points are mean values of the atomic ratios measured at specific locations inside the crack. Locations closer to the surface of the sample (crack opening) had smaller values, whereas locations in the deeper parts of the crack were marked with higher numbers. A similar relation was also observed for concrete healed in marine environments [10].

The presence of phosphorus in the healing products was related to the chemical composition of the applied retarder, which is based on a sodium metaphosphate. The retarding action was based on the bonding of calcium ions Ca^2+^ with phosphate anions and the formation of unstable calcium hydrogen phosphate. The lack of Ca^2+^ ions in the solution inhibited the formation of CSH, leading to a delayed initial setting time [39]. The dissolution of the calcium hydrogen phosphate was followed by the formation of less soluble phases, i.e., secondary CSH gel and calcium hydroxyapatite (HAP) [39]. However, when the phosphate concentration was sufficiently high, the delay could be shortened due to the precipitation of the hydroxyapatite [40]. Those two processes, i.e., the adsorption of phosphates on cement grains and the formation of the HAP, were likely to contribute to the observed efficient crack closure of the samples in Exposure 2. In fact, both CSH as well as calcite present inside the crack were shown earlier to be efficient in the removal of phosphates from phosphate-contaminated solutions [41,42,43]. This could further support the hypothesis, assuming the formation of a calcium phosphate compound inside the crack. HAP, as a healing product, could be considered desirable due to its significant stability and insolubility, especially in comparison to calcium hydroxide [44]. Cracks observed in the healing products (e.g., Figure 14b) could be related to the development of shrinkage during the conversion of a HAP precursor, which was presumably amorphous calcium phosphate (ACP), into the HAP [45]. Earlier, the HAP was observed not to form homogenously, but to be preceded by the growth of precursors [46,47]. Chemical compositions of possible phases formed inside the crack are listed in Table 3.

The type of a phosphate-retarding agent is also important in the precipitation mechanism. Earlier studies found that the retarding effect differs with respect to the thickness of the phosphate layers adsorbed on the cement grains [48]. The monophosphate compounds led to the precipitation of the calcium-based phosphate close to the cement particles. However, polyphosphates also contributed to the growth of a Ca-phosphate complex [48].

The precipitation of calcium phosphate compounds could also explain the relatively low pH observed in the case of Exposure 2 (Figure 12a). The mixing of the calcium and phosphate solutions led to a quick decrease of the pH value within just few minutes after mixing, followed by its slow decrease before reaching a stable level after 2 h [49].

#### 3.1.4. Calcium Ions and Silica Microparticles

**Figure 16:**
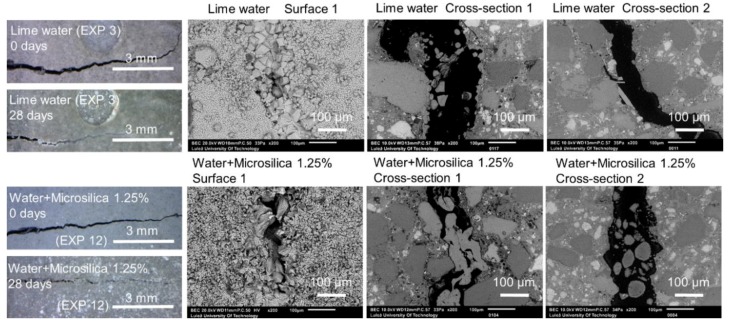
Representative images of the surface and cross-sections of the specimens healed in Exposures 3 (lime water) and 11 (water and 1.25%w microsilica particles).

Crack healing for Exposures 3 and 11 using limewater and water with micro silica particles, respectively, is shown in Figure 16. All exposures produced externally visible crack healing. The SEM-BSE investigation showed internal healing for the exposure with a mixture of water with micro silica (EXP 11). The exposure to lime water (EXP 3) did not result in internal healing.

**Figure 17:**
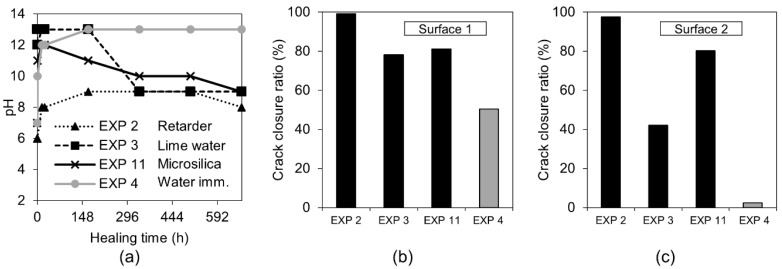
(**a**) pH changes in time for Exposures 4, 2, 3, and 11; crack closure ratio for Exposures 4, 2, 3, and 12 on (**b**) Surface 1 (**c**) Surface 2.

The calculation of the crack closure ratio based on the optical microscope images showed a general increase in surface healing, in Surface 1 and Surface 2, in comparison to the reference Exposure 4 (water immersion) (Figure 17b,c). Others reported similar results for the limewater exposure [24]. None of the three exposures reached the crack closure efficiency observed for Exposure 2, which contained the retarder. All measured pH values initially increased and subsequently decreased for Exposure 3 (limewater) and Exposure 11 (microsilica) (Figure 17a). A stable pH value of nine was reached after approximately two weeks of exposure.

**Figure 18:**
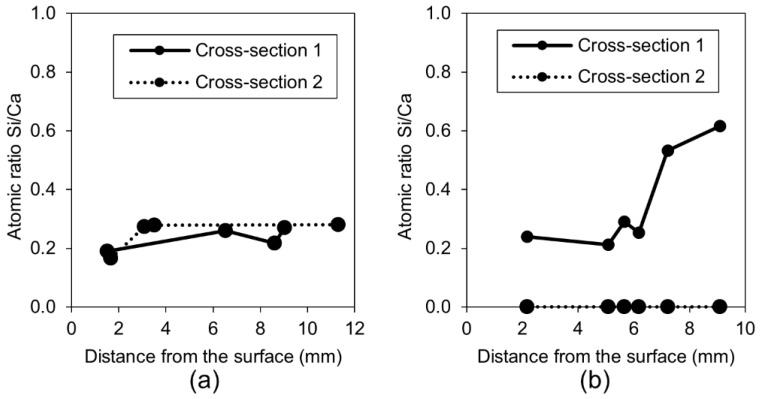
Si/Ca atomic ratios: (**a**) Exposure 3 (Limewater) and (**b**) Exposure 11 (Microsilica).

**Figure 19:**
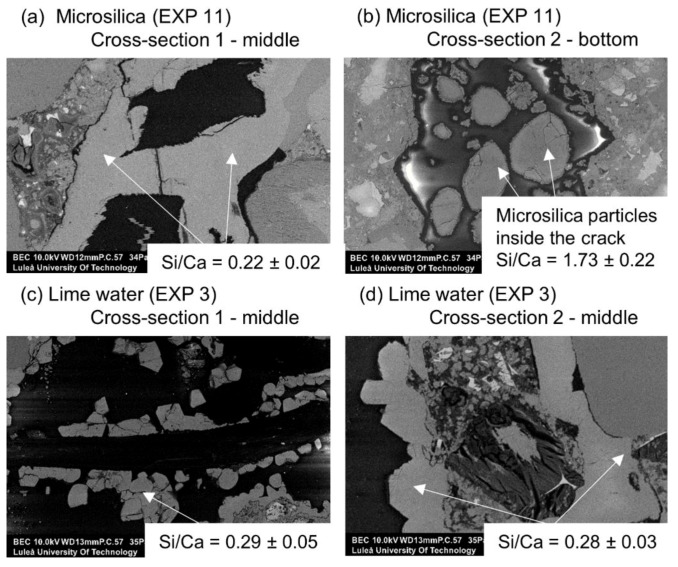
(**a**) Self-healing products at Cross-section 1 in the middle of the crack (EXP 11, SEM BSE image 400×), (**b**) self-healing products at Cross-section 2 at the bottom of the crack (EXP 11, SEM BSE image 400×), (**c**) self-healing products at Cross-section 1 in the middle of the crack (EXP 3, SEM BSE image 500×), and (**d**) self-healing products at Cross-section 2 in the middle of the crack (EXP 3, SEM BSE image 1000×).

The chemical composition of the self-healing phases inside the crack for Exposure 3 (limewater) did not show similarities with Exposure 2 (retarder) in regards to the Si/Ca ratio (Figure 18a). The Si/Ca for Exposure 3 was relatively stable along the crack length on both measured cross-sections. The morphology of both the external and internal healing products indicated that the majority of the precipitated compounds were a combination of the calcium carbonate and Portlandite mixed with the CSH (Figure 19c,d). The additional calcium ions introduced to the crack by the saturated lime solution (EXP 3) presumably enhanced the internal self-healing in comparison to reference Exposure 4 (water immersion). Exposure 11, which contained a mixture of water with microsilica, showed very high external self-healing with a dense calcite structure that filled the crack. However, internally, only single silica particles were found without the formation of any self-healing products on their surface, indicating the insufficient efficiency of this Exposure (Figure 19a,b).

### 3.2. Strength Recovery

**Figure 20:**
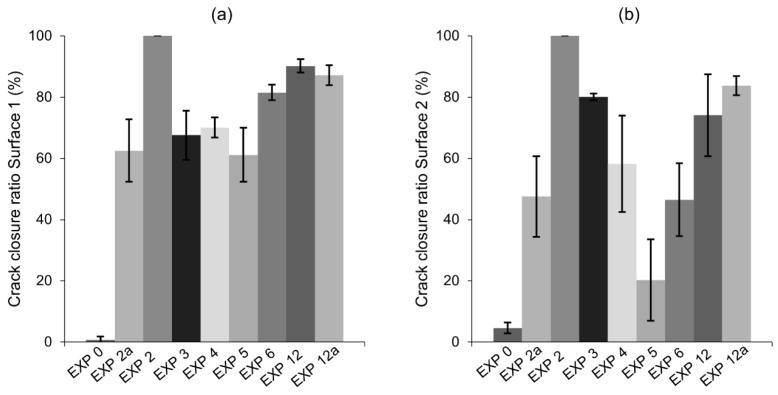
Crack closure of selected exposures: (**a**) Surface 1 and (**b**) Surface 2.

The flexural strength recovery was studied for the exposures that gave the most satisfactory self-healing observations, i.e., Exposure 2 (Retarder), Exposure 3 (Saturated limewater), Exposure 4 (reference, water immersion), Exposure 5 (water evaporation), and Exposure 11 (water with micro silica), as well as Exposure 0 (cured in the air) for comparison. In addition, different amounts of retarder and silica fume were used to verify the effects of the amount of an active ingredient on the healing efficiency. Exposure 2a contained 7 wt%, while Exposure 11a contained 2.5 wt% of microsilica in water. In addition to the strength recovery test, the optical microscope based estimation of the crack closure ratio was done. Data were collected from five samples for each exposure. The crack closure observations confirmed a high external self-healing efficiency of the selected exposures on Surface 1 of the crack (Figure 20a). However, Surface 2 (the crack opening) was difficult to heal (Figure 20b), with only Exposure 2 and 11 achieving high crack closure ratio. High standard deviation values for Surface 2 also indicated the randomness of this process.

**Figure 21:**
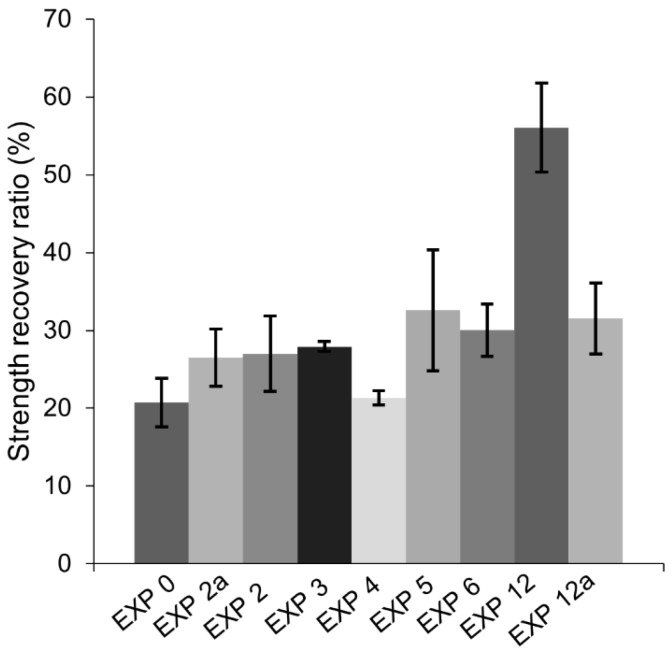
Strength recovery for selected exposures.

The flexural strength recovery results (Figure 21) demonstrated that only Exposure 11 (microsilica 1.25%) achieved a moderate strength regain, reaching approximately 60%. None of the remaining specimens presented any meaningful strength recovery, as the values obtained were equal to the strength recovery of the samples cured in air. This might suggest that even satisfactory internal self-healing of the crack, as in the case of Exposure 2 (retarder), might not be sufficient to regain flexural strength.

## 4. Discussion

The summary of the observed effects of both internal and external self-healing for each applied exposure is presented in Table 4.

In general, water-related exposures (Exposures 4–8) did not give satisfying self-healing results despite the application of different cycles or water volumes. Similar results were observed earlier [18]. A higher ion concentration, expected in the case of a smaller amount of water present inside the crack (i.e., Exposures 7 and 8), did not support the healing process. However, it might be related to a generally small amount of ions being transported to the crack from the cement matrix due to a dense microstructure of the binder matrix resulting from a low water-to-cement ratio [25]. Another problem, contributing to a lack of the internal self-healing products might be the precipitation of the hydration products on unhydrated cement grains, which then block the further hydration and thus diminish the closing of the cracks [50]. Temperature cycles (Exposures 9 and 10) were equally inefficient. However, the extensive external growth of Ettringite in the case of the applied cycles, 24 h/20 °C and 24 h/5 °C (Exposure 10), indicated the possibility of increasing the crack closure by Ettringite. The preferential formation of Ettringite could be induced by the optimization of the mix using special cementitious binder and/or by extending the low temperature part of the cycle. The long-term effects of low temperature on hydration are positive with respect to the strength and density of hydration products [27], therefore the self-healing of cementitious materials in low temperature should be further studied.

**Figure 22:**
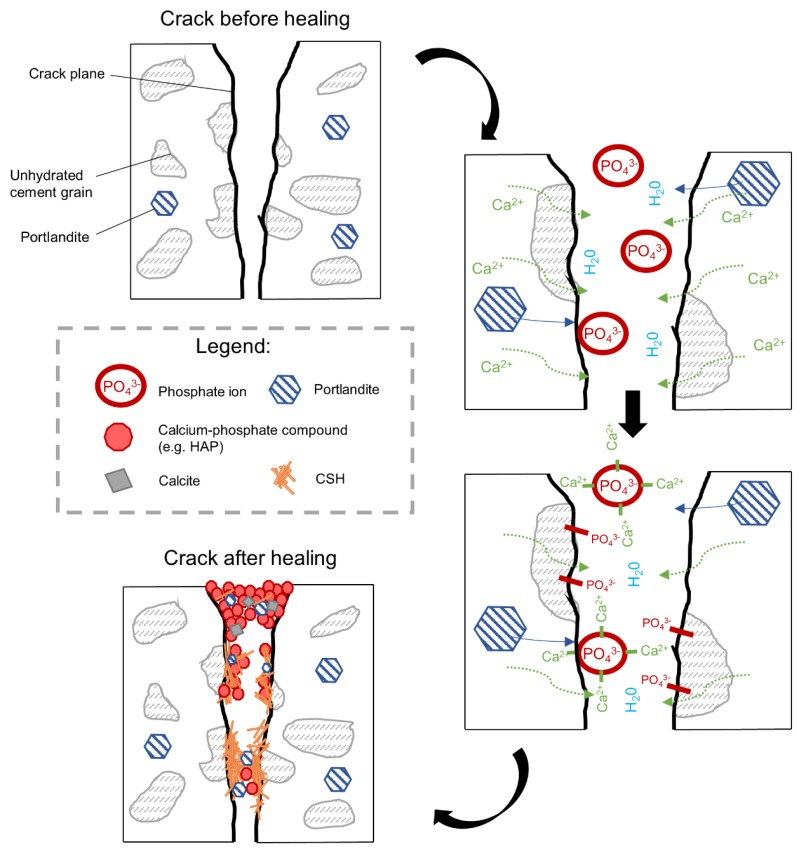
A possible self-healing mechanism for samples exposed to phosphate based retarder conditions.

The addition of the phosphate-based retarding admixture (Exposure 2) demonstrated the highest crack closure, both internally and externally. A proposed mechanism behind this process is shown in Figure 22. The chemical mechanism can be assumed, based on the results obtained from SEM and EDS analyses, and is connected to two different processes. The retarding effects are related to the adsorption of phosphate ions on the surface of unhydrated cement grains resulting in a slower hydration process, which prevents the quick formation of a dense encapsulating shell. A higher observed amount of the formed CSH inside the crack [47], particularly deep inside the sample, where the concentration of the ions from cement binder is higher, supports that mechanism. Another process is connected to the precipitation of calcium-phosphate compounds formed by the chemical reaction of the calcium ions transported from the binder matrix (e.g., Portlandite) and the phosphate ions from the retarder. A higher amount of the phosphorus phase at the top of the crack could indicate that the majority of precipitates formed in this part of the crack are calcium–phosphate compounds. This process might be responsible for the higher crack closure efficiency observed at a higher concentration of the retarder (Figure 20, Exposure 2 and 2a). A lack of strength recovery (Figure 21) could further support that mechanism as calcium–phosphate compounds have lower strength than the CSH phase.

The highest strength recovery and a very good crack closure ratio was achieved due to the immersion in water mixed with microsilica particles (Exposure 11). It can be assumed that the microsized silica particles served as nucleation sites for the formation of the CSH, as well as calcium carbonate and Portlandite. All those phases provided a good self-healing efficiency and enabled the formation of load bearing phases. Similar effect can be attributed to the presence of the PVA fibers.

## 5. Conclusions

The aim of this paper was to investigate the effects of various self-healing solutions on the efficiency of an external and internal crack closure. Four groups of exposures were tested: Different water immersion regimes, the effects of temperature, the presence of the hydration rate that modified admixtures, and additional ions/particles in the self-healing solution. The main conclusions were formulated as follows:

- The water-related exposures did not give satisfying self-healing results despite the application of different cycles or water volumes. A higher ion concentration, expected in the case of a smaller amount of water present inside of the crack, did not support the healing process;
- The addition of the phosphate-based retarding admixture demonstrated the highest crack closure both internally and externally. Phosphate ions were found to contribute to the filling of the crack, most likely by preventing the formation of a dense shell composed of the hydration phases on the exposed crack by unhydrated cement grains. Phosphate ions also caused the formation of calcium–phosphate based compounds;
- The highest strength recovery and a very good crack closure ratio was achieved by immersion in water mixed with microsilica particles. The micro sized silica particles presumably served as nucleation sites for the formation of the CSH, calcium carbonate, and Portlandite.

The following limitations of the performed study can be listed and considered in future research:

- The applied chemicals were of an industrial/technical grade, which generated a number of different factors that should be considered. Even though this is the case in real-life concrete applications, in order to fully understand the mechanisms of the autogenous self-healing, higher purity chemical substances should be used to separate the variables;
- The SEM evaluation should be performed on a larger number of specimens in order to enable quantitative evaluation of the internal self-healing products. It is especially crucial due to the strong effects of the crack width and shape;
- In order to fully confirm the chemical composition of the precipitated self-healing phases, the performed elemental analysis should be complemented by an evaluation of the mineralogical composition using, for example an X-ray powder diffraction (XRD). Larger specimens should be tested, thus increasing the amount of the analyzed phases;
- As the used retarding admixture significantly enhanced the self-healing efficiency, different kinds of retarders could be compared;
- Mineralogical composition, solubility, durability, and other physical properties of the calcium phosphate phase should be verified with respect to its full scale applicability;
- The combination of different exposures, which showed the highest self-healing efficiency, could be tested, e.g., a retarding admixture together with microsilica particles.

## Figures and Tables

**Table 1:** Chemical composition of the Portland cement used.

Chemical Analysis	Mean Value (%)
CaO	63.30
SiO_2_	21.20
Al_2_O_3_	3.40
Fe_2_O_3_	4.10
MgO	2.20
Na_2_O	0.18
K_2_O	0.56
SO_3_	2.70
Cl	<0.01
Loss of ignition	2.50
Water soluble Cr^6+^	<2 mg/kg
Na_2_O-eq.	0.55

**Table 2:** List of exposure conditions applied with justifications.

Exposure	Abbreviation	Justification
Air	EXP 0	Non-healed samples
Deionized water mixed with Accelerator in proportions 3:1 (immersion)	EXP 1	Increasing the rate of hydration process inside the crack; possibly faster healing; different composition of hydrates [26,27,28]
Deionized water mixed with Retarder in proportions 3:1 (immersion)	EXP 2	Slowing down the hydration—more hydrates can precipitate on the surface of unhydrated cement grains [27,29]
Saturated lime water immersion	EXP 3	More Ca^2+^ ions in the solution, higher pH
Deionized water immersion	EXP 4	Reference exposure
Deionized water immersion with cyclic evaporation (72 h cycle)	EXP 5	Changing of the water regime by introducing the cycles of evaporation as well as different volume of water in order to modify the concentration of ions inside the crack
Dry/wet (deionized water) cycles; 24 h dry and 24 h immersion in water	EXP 6	
Deionized water immersion up to 1 mm height of the sample	EXP 7	
Deionized water immersion up to 5 mm height of the sample	EXP 8	
Water immersion temperature cycle 24 h/20 °C and 24 h/40 °C	EXP 9	Increasing/decreasing the rate of the hydration process as well changing the hydration products composition. Possible ettringite formation leading to a higher strength regain in case of lower temperature [30,31]
Water immersion temperature cycle 24 h/20 °C and 24 h/5 °C	EXP 10	
Deionized immersion with microsilica particles 1.25 %w	EXP 11	Providing the nucleation sites inside the crack for the self-healing products

**Table 3:** Calcium phosphate phases [46].

Phase	Composition	Ca/P
Brushite (DCPD)	CaHPO_4_ 2H_2_O	1.00
Monetite (DCPA)	CaHPO_4_	1.00
Octacalcium phosphate (OCP)	Ca_4_H(PO_4_)_3_ 2.5H_2_O	1.33
Whitlockite/tricalcium phosphate (TCP)	Ca_3_H(PO_4_)_2_	1.50
Hydroxyapatite (HAP)	Ca_5_(PO_4_)_3_OH	1.67
Amorphous calcium phosphate (ACP)	-	-

**Table 4:** Summary of the results for all exposures.

Exposure	Abbreviation	External Self-Healing	Internal Self-Healing
Deionized water mixed with Accelerator in proportions 3:1 (immersion)	EXP 1	Very limited crack closure; Several calcite crystals of various shapes	Almost no healing; Several microns thick calcite layer under the surface, inside—few thicker deposits of calcite mixed with CSH (Si/Ca = 0.19)
Deionized water mixed with Retarder in proportions 3:1 (immersion)	EXP 2	The crack almost completely healed; calcium phosphate compounds on the surface with some amount of sodium originating from the self-healing mixture	Very high internal crack closure; Calcium phosphate compounds as well as CSH; Si/Ca and Ca/P increasing with crack depth
Saturated lime water immersion	EXP 3	Very good external self-healing; dense layer of calcite crystals present at the surface	Almost no internal self-healing; few self-healing products with average Si/Ca of 0.3
Deionized water immersion	EXP 4	Very limited external healing, ettringite and calcite crystals filling the crack	Ettringite visible close to the surface; no internal self-healing
Deionized water immersion with cyclic evaporation (72 h cycle)	EXP 5	Some crack closure; bigger calcite crystals covering the crack	Layer of calcite inside of the sample closure to the surface, few self-healing products in deeper parts of the crack
Dry/wet (deionized water) cycles 24 h/24 h	EXP 6	Almost no external self-healing with small calcite crystal layer at the surface	No internal self-healing except for few healing products deposited on the PVA fibers surface
Deionized water immersion up to 1 mm height of the sample	EXP 7	Minimal external healing, ettringite present; no noticeable differences between exposure 8 and 9	Ettringite visible close to the surface; no internal self-healing
Deionized water immersion up to 5 mm height of the sample	EXP 8		
Water immersion temperature cycle 24 h/20 °C and 24 h/40 °C	EXP 9	Very limited external healing, calcite crystals filling the crack	Hardly any internal healing with only single calcite crystals
Water immersion temperature cycle 24 h/20 °C and 24 h/5 °C	EXP 10	Efficient external crack closing; calcite crystals inside the crack as well as thick layer of ettringite	
Deionized immersion with microsilica particles 1.25%w	EXP 11	Very high external self-healing with densified calcite structure filling the crack	Agglomerates of microsilica particles inside the crack without self-healing products.
